# Two-Component Response Regulators CitT, YvcP, and YycI Differentially Control Pectin and Hemicellulose Degradation in Degumming of Ramie Fibers by *Bacillus subtilis* Strain 168

**DOI:** 10.3390/polym17182473

**Published:** 2025-09-12

**Authors:** Qi Yang, Shihang Ma, Lifeng Cheng, Xiang Zhou, Guoguo Xi, Chen Chen, Zhenghong Peng, Yuqin Hu, Si Tan, Shengwen Duan

**Affiliations:** Institute of Bast Fiber Crops, Chinese Academy of Agriculture Sciences, Changsha 410205, China; yangqi@caas.cn (Q.Y.); ma764713068@126.com (S.M.); chenglifeng@caas.cn (L.C.); 821012430018@caas.cn (X.Z.); xiguoguo@caas.cn (G.X.); buct_cc@163.com (C.C.); pengzhenghong@caas.cn (Z.P.); 82101232017@caas.cn (Y.H.); 15116005853@163.com (S.T.)

**Keywords:** two-component system response regulator, *Bacillus subtilis* strain 168, microbial degumming of ramie, pectin degradation, hemicellulose degradation

## Abstract

Exploring the metabolic regulatory mechanisms of bacteria for ramie degumming and constructing more efficient engineered strains are preferred strategies to solve the technical bottleneck of high residual gum content in fibers. *Bacillus subtilis* strain 168, an advantageous bacterium for microbial degumming, was previously found to significantly up-regulate the expression of bast two-component system (TCS) response regulators CitT, YvcP, and YycI when using ramie as the sole carbon source. In this study, the genes encoding CitT, YvcP, and YycI proteins were knocked out and compared the effects between these gene knockouts and the original strain on the degumming efficiency. The aim was to identify the key TCS response regulators that significantly affect degumming efficiency and to explore the functions of these different response regulators. The results demonstrated that knockout of *citT*, *yvcP*, or *yycI* genes significantly reduced degumming efficiency. Specifically, CitT protein primarily regulated the degradation of pectin, YvcP protein mainly regulated the degradation of hemicellulose, and YycI protein was involved in the regulation of both pectin and hemicellulose degradation. Notably, the absence of CitT protein caused the most significant reduction in degumming efficiency. These findings provide valuable insights into the construction of engineered strains with high degumming efficiency for ramie fibers.

## 1. Introduction

Ramie, commonly known as “China grass”, is a highly distinctive economic crop in China and an important source of natural fibers. It is widely used in various fields, including textiles, rail transit, aerospace, and medical supplies [1,2]. Obtaining high-quality ramie fibers is a crucial prerequisite for the development of ramie industry. In addition to fibers, the bast of ramie contains 25–35% of bonded non-cellulosic substances (commonly referred to as “gum”), which are composed of pectin, hemicelluloses (such as xylans and mannans) and a small amount of lignin [3,4]. The removal of these bonded non-cellulosic substances (the “degumming” process) is essential for obtaining ramie fibers. Degumming is primarily executed by using two methods: chemical degumming and biological degumming. The chemical degumming method widely used in industry is costly, energy-intensive, and severely pollutes the environment. Moreover, it results in low fiber yield and poor fiber quality. The difficulty in degumming is the primary factor restricting the development of the ramie industry. Biological degumming can save a significant amount of chemical materials and energy and greatly reduce the environmental pollution caused by degumming wastewater, and it can also produce higher-quality fibers [5]. There are two approaches involved in biological degumming: enzymatic degumming and microbial degumming. Enzymatic degumming involves the heterologous expression of functional enzymes (such as pectinase, xylanase and so on) for ramie degumming [6,7,8,9]. However, enzymatic degumming has several drawbacks, including the challenge of purifying degumming enzymes, high costs, and immature techniques for enzyme immobilization and recovery. Therefore, it is difficult to apply enzymatic degumming to large-scale industrial production. Microbial degumming utilizes the metabolism function of degumming microorganism (mostly bacteria) to biologically transform the gum into absorbable carbon source. This technology has the advantages of being high-quality, efficient, energy-saving, and environmentally friendly, and it is an important direction for research on ramie degumming [10]. Microbial degumming, which includes both anaerobic and aerobic processes, is highly dependent on the use of efficient bacterial strains [11,12]. Currently, most ramie-degumming bacteria are wild-type strains isolated from natural environments, such as *Bacillus pumilus* DKS1, *Bacillus* sp. strain N16-5, and *Bacillus* sp. Y1 [13,14,15], indicating that degumming-capable microorganisms are predominantly found within the genus *Bacillus*. However, wild-type strains often exhibit limited degumming efficiency, leading to high residual gum content in the fibers and hindering industrial-scale application. A crucial strategy for enabling large-scale microbial degumming is the development of enhanced engineered strains through targeted genetic modifications. For instance, synergistic improvement of xylanase activity combined with optimized pretreatment has been shown to significantly enhance the degumming efficiency of ramie bast fibers [12]. However, a few engineered strains have been developed to suitable for large-scale industrial degumming production to date. The reason for this is that the metabolic regulation mechanisms of microbial degumming are not yet clear, and there is a lack of theoretical guidance for the targeted modification of wild-type degumming strains. Therefore, elucidating the complex, precise, and rigorous metabolic regulation mechanisms of microbial degumming process is an important prerequisite for the construction of highly efficient ramie degumming engineered strains.

Hundreds of ramie degumming strains have been isolated from natural environment, among which the genus *Bacillus* accounts for more than 70%, and most of them are *Bacillus subtilis*. Investigating the metabolic regulation mechanisms of microorganisms in ramie degumming using *B. subtilis* as the target species is of significant guiding importance for the construction of efficient engineered strains. *B. subtilis* strain 168 is the model strain of the genus *Bacillus* and a representative strain for the study of the physiology and genetics of Gram-positive bacteria. There have been reports of heterologous expression of pectinase from *B. subtilis* strain 168 for ramie degumming [16,17], indicating the potential of this strain for microbial degumming of ramie. The essence of microbial degumming is the complex physiological and metabolic process that occurs when degumming bacteria utilize the gum in ramie bast as a carbon source, which triggers a series of metabolic regulatory responses in the bacteria. The regulation of carbon source metabolism is directly linked to the induction mechanisms for the synthesis of respective glycosyl hydrolases, particularly in the presence of diverse carbohydrates [18,19]. Glucose is the most commonly used rapidly available carbon source and is often used as a control for other carbon sources in the study of microbial metabolic regulation mechanisms [20,21]. Our preliminary research found that *B. subtilis* strain 168 can survive under the culturing conditions where ramie bast is the sole carbon source, and its growth characteristics are significantly different from those when glucose is used as the carbon source [22]. Comparative proteomics studies revealed that the difference in carbon sources led to significant differences in the abundance of more than 360 proteins in *B. subtilis* strain 168, with the functions of these proteins mainly involving carbon metabolism and two-component signal transduction systems [22].

The two-component system (TCS) is the predominant signaling system in bacteria. TCS is the primary mechanism by which bacteria respond to changes in their environment and regulate most physiological processes, including growth and nutrient metabolism [23]. TCS are comprising a histidine kinase and a response regulator, the kinase relaying environmental signals to the response regulator, after the activation of response regulator, it initiates the transcription, translation, and post-translational modifications of a series of related genes, thereby exerting its biological regulatory functions [24]. Our preliminary research identified that among all the detected TCS response regulators, only CitT, YvcP, and YycI exhibited significant differences in expression abundance. These response regulators were significantly up-regulated under conditions where ramie bast was used as the sole carbon source (detected only under this growth condition, with qRT-PCR validation also showing an up-regulation trend at the transcriptional level) [22]. Based on these findings, we hypothesize that the differential expression of the TCS response regulators CitT, YvcP, and YycI in *B. subtilis* is closely related to the unique carbon source (ramie bast) in the culturing environment and may be key potential factors in regulating ramie degumming.

The response regulators CitT, YvcP, and YycI in *B. subtilis* strain 168 are derived from the two-component systems CitST, YvcPQ, and WalRK, respectively. Studies have indicated that these regulators are primarily involved in the regulation of multiple physiological processes, including citrate metabolism, antibiotic stress, spore germination, and phosphate metabolism [25,26,27]. However, it remains unclear which specific TCS response regulator—CitT, YvcP, or YycI—plays the most critical role in regulating the degumming process in *B. subtilis*. To our knowledge, no comparative functional analysis of these regulators in ramie degumming has been reported to date. The aim of this study is to evaluate the impact of these target response regulators (CitT, YvcP, or YycI) on the degumming efficiency of ramie and identify the key TCS response regulator involved in ramie degumming by constructing gene knockout strains and analyzing the degummed ramie fibers.

## 2. Materials and Methods

### 2.1. Functional Prediction of Response Regulators by Bioinformatic Analysis

To investigate protein–protein interactions and their functional associations, the proteins CitT, YvcP, and YycI were analyzed using the STRING database (https://cn.string-db.org/). The analysis was performed with *B. subtilis* as the organism of interest, and the highest confidence score was set at 0.9.

### 2.2. Experimental Materials

Ramie was sourced from Yuanjiang City, Hunan Province, China. The experimental material used was the first crop of Zhongzhu No.1, obtained from the Yuanjiang Comprehensive Experiment Station of the China Agricultural Research System (Bast Fiber Crops). The ramie bark was manually stripped from the core, dried and stored without mildewing.

### 2.3. Strains

*B. subtilis* strain 168 was kindly provided by Professor Qingshu Liu from the Hunan Institute of Microbiology, Changsha, China. The *B. subtilis* Δ*eglS* mutant was constructed in our laboratory. The *B. subtilis* strain 168 Δ*eglS*, which is deficient in cellulase, was used for degumming of ramie [28]. This strain was defined as the original strain for further genome editing. The genes *citT*, *yvcP*, and *yycI*, encoding the proteins CitT, YvcP, and YycI, respectively, were knocked out using the CRISPR-Cas9 system [29]. The knockout was performed by Forhigh Biotech (Hangzhou, China) Co., Ltd. (http://www.forhighbio.com/), and confirmed in our laboratory by PCR using three pairs of primers: citTF/citTR, yvcPF/yvcPR, and yycIF/yycIR. Primer pairs citTF (TTGATTCACATCGCGATTGC) and citTR (CTAATCCGCCGCCAAATA) could amplify full length of gene *citT*, primer pairs yvcPF (GTGTATCGGATTTTGCTTGTGG) and yvcPR (TTAACATTCCGCTTCATCCTTC) could amplify full length of gene *yvcP*, primer pairs yycIF (GTGGAGTGGAATAAGACAAAATC) and yycIR (TCATTGATCTGTATCTAAAATCGTAC) could amplify full length of gene *yycI*. The PCR reaction system consisted of 0.2 µL of genomic DNA, 14.8 µL of double-distilled water, 2 µL of 10× PCR buffer, 1.8 µL of dNTP mixture, 0.5 µL of each primer, and 0.2 µL of rTaq DNA polymerase. The PCR program was as follows: initial denaturation at 94 °C for 5 min, followed by 30 cycles of 94 °C for 30 s, 60 °C for 30 s, and 72 °C for 1 min (or 1.5 min), with a final extension at 72 °C for 10 min and holding at 20 °C. The confirmed mutant strains were designated as Δ*citT*, Δ*yvcP*, and Δ*yycI.*

### 2.4. Growth Conditions and Profiles

All strains were initially activated in 20 mL of LB liquid medium at 37 °C with shaking at 180 rpm for 12 h. Subsequently, 0.5 mL of the culture was transferred to 50 mL of LB liquid medium and incubated at 37 °C with shaking at 280 rpm until the late exponential growth phase (approximately 6–8 h, and OD_600_ nm = 0.7–0.8). The seed culture was then transferred to a degumming system containing ramie bast, with an inoculation volume of 10%. The system was incubated at 35 °C with shaking at 280 rpm for 25 h. The surface morphology of the ramie fibers after degumming was analyzed using a scanning electron microscope (SEM, Hitachi SU-3500,Hitachi High-Tech Corporation, Tokyo, Japan). The degumming system was prepared in a 500 mL flask containing 10 g of ramie bast and 100 mL of deionized water, sterilized at 121 °C and 103.4 kPa for 20 min. The growth profile of *B. subtilis* in the defined fermentation medium was monitored by measuring the optical density at 600 nm (OD_600 nm_), with strains cultured in LB medium serving as controls.

### 2.5. Post-Treatment and Chemical Component Analysis of Ramie Fibers

The post-treatment of ramie fibers was performed as previously described [28]. Briefly, all samples were thoroughly washed to remove residual degraded gum still adhering to the fibers after degumming, and then dried at 60 °C until constant weight was achieved for subsequent determination of weight loss ratio and residual gum content. The weight loss ratio (*M*) was calculated using the following Formula (1):

(1) $$M=\frac{M_{0}-M_{1}}{M_{0}}\times 100\%$$

where *M*_0_ and *M*_1_ were the weights of the ramie fibers before and after degumming, respectively.

The residual gum content (*W*) was determined as previously described [30]. Ramie fiber samples were dried at 60 °C to a constant weight (*W*_0_). The samples were then boiled in a fixed condenser-Allihn apparatus with 200 mL of water and 20 g/L of NaOH for 2 h. The residues were cleaned by washing with water three times and then dried to a constant weight (*W*_1_) at 60 °C. The residual gum content was calculated using the following Formula (2):

(2) $$W=\frac{W_{0}-W_{1}}{W_{0}}\times 100\%$$

where *W*_0_ and *W*_1_ were the weights of the degummed ramie fibers treated with different strains before and after alkaline boiling, respectively.

The single-fiber breaking strength of the treated ramie fibers was assessed as before [28]. The chemical components of the ramie fibers before and after degumming were analyzed according to the national standard of China [31]. The degradation rates (*P*) of lignin, pectin and hemicellulose were calculated as previously described [32], and using the following Formula (3):

(3) $$P=\frac{P_{0}-P_{1}}{P_{0}}\times 100\%$$

where *P*_0_ and *P*_1_ were the content of lignin, pectin, or hemicellulose in ramie fibers without and with microbial treatment, respectively.

### 2.6. Enzymatic Activity Analysis

Samples were collected from both the original and mutant strains cultured in LB broth (8 h) or the ramie bio-degumming system (5 h) during the late exponential growth phase. The cultures were centrifuged at 6000 rpm for 5 min at 4 °C. The supernatants were then filtered through 0.22 µm Millipore membranes on ice and subsequently used for enzymatic activity assays. The activities of pectinase (BC2635, Beijing Solarbio Science & Technology Co., Ltd., Beijing, China), neutral xylanase (BC2595, Beijing Solarbio Science & Technology Co., Ltd., Beijing, China), alkaline xylanase (BC3615, Beijing Solarbio Science & Technology Co., Ltd., Beijing, China), acidic xylanase (BC2605, Beijing Solarbio Science & Technology Co., Ltd., Beijing, China), and β-mannanase (ADS-F-MAN003, Jiangsu Aidisheng Biological Technology Co., Ltd., Yancheng, China) were measured using ultraviolet spectrophotometry, in accordance with the manufacturers’ instructions.

### 2.7. Statistical Analysis

All quantitative data presented in this study were obtained from three independent biological replicates, which account for the inherent biological variability. Each biological replicate consisted of three technical replicates to ensure the precision of individual measurements. The data are expressed as the mean ± standard deviation (SD). The SD values, which quantify the dispersion of data points around the mean from the biological replicates, were calculated using the descriptive statistics function of the data analysis toolpack in Microsoft^®^ Excel.

## 3. Results and Discussion

### 3.1. Functional Prediction of TCS Response Regulators

**Figure 1:**
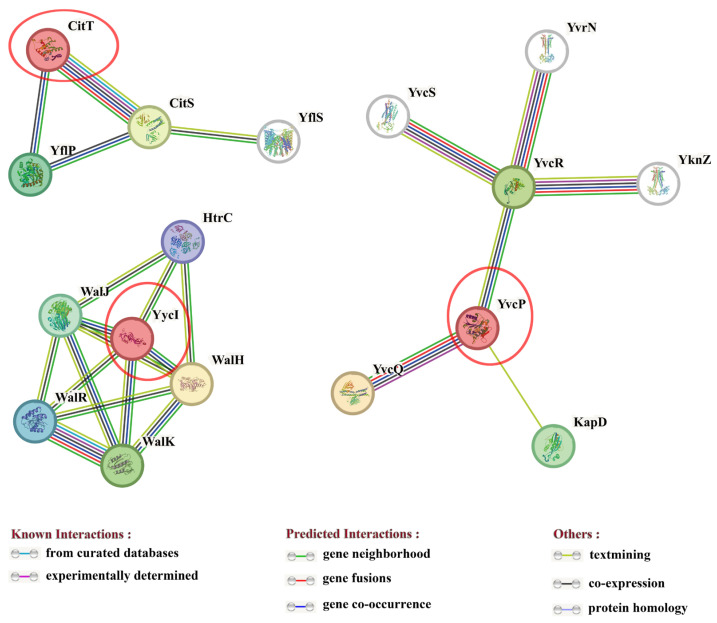
Network analysis of protein–protein interactions and functional associations. The predicted functional partners of CitT, YvcP, and YycI were analyzed using the STRING software version 12.0 with the highest confidence score (0.9). Red circles indicate the response regulator proteins CitT, YvcP, and YycI. Network nodes represent different proteins, with node colors used solely for visual distinction. Filled nodes denote proteins with known or predicted three-dimensional (3D) structures.

To further elucidate the regulatory functions of the response regulator proteins CitT, YvcP, and YycI in the degumming of ramie by *B. subtilis*, the interactions and regulatory networks among these proteins were analyzed by STRING database version 12 (Figure 1). It revealed that CitT, YvcP, and YycI do not interact with each other directly.

CitT was found to interact with three proteins: the kinase CitS, and the proteins YflS and YflP. YflS has been reported to play a role in the regulation of *B. subtilis* metabolism when using malate as a carbon source [33], while the function of YflP remains to be elucidated. CitT is involved in the metabolic regulation of carbon sources such as citrate [34]. Based on these findings, it hypothesized that CitT and its associated proteins are mainly involved in the regulation of carbon source metabolism in *B. subtilis*.

YycI was found to interact with five proteins: HtrC, WalJ, WalH, WalR, and WalK. HtrC is closely related to spore germination in *B. subtilis*, and its absence affects spore formation [35]. WalJ is involved in cell division and cell wall metabolism in *B. subtilis* [36]. But the function of WalH in *B. subtilis* is currently unknown. WalR and WalK form a two-component system WalRK, with WalR being involved in the response of *B. subtilis* to heat stress [37], and the WalRK two-component system is associated with the homeostasis of cell wall hydrolases [38]. Based on these findings, it speculated that YycI and its associated proteins are mainly involved in cell wall metabolism and survival regulation under environmental heat stress conditions in *B. subtilis*.

YvcP was found to interact with six proteins: YvcQ, KapD, YvcR, YknZ, YvcS, and YvrN. YvcP and YvcQ form a two-component system that, together with KapD and YvcR, regulates resistance to bacteriocins in *Bacillus thuringiensis* [26]. YknZ is a component of the four-protein transporter YknWXYZ and plays a protective role in sporulation-delaying-protein-induced killing of *B. subtilis* [39]. YvrN is involved in metal ion uptake in *B. subtilis* [40]. But the function of YvcS has not been reported. Based on these findings, it proposed that YvcP and its associated proteins are mainly involved in stress resistance, self-protection, and metal ion uptake in *B. subtilis*.

As the microbial degumming of ramie is a complex metabolic process, further investigation is warranted to determine which of the three response regulator proteins—CitT, YvcP, and YycI—serves as the key regulatory protein in the degumming process.

### 3.2. Identification of Mutant Strains and Analysis of Growth Trends

**Figure 2:**
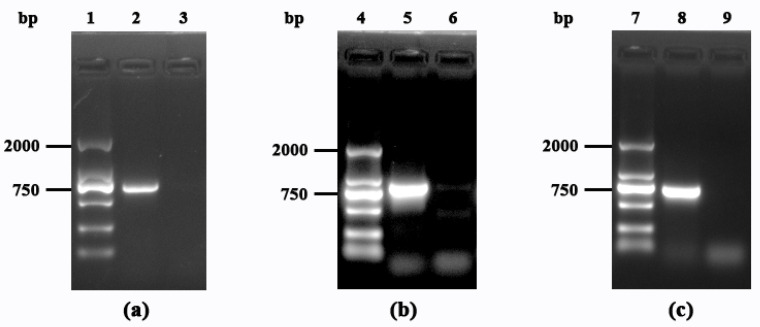
PCR identification of the mutant strains Δ*citT*, Δ*yvcP*, and Δ*yycI*. (**a**) PCR amplification of the gene *citT*. Lane 1: DNA marker DL 2000; Lane 2: original strain; Lane 3: Δ*citT* mutant strain; (**b**) PCR amplification of the gene *yycI*. Lane 4: DNA marker DL 2000; Lane 5: original strain; Lane 6: Δ*yycI* mutant strain; (**c**) PCR amplification of the gene *yvcP*. Lane 7: DNA marker DL 2000; Lane 8: original strain; Lane 9: Δ*yvcP* mutant strain.

**Figure 3:**
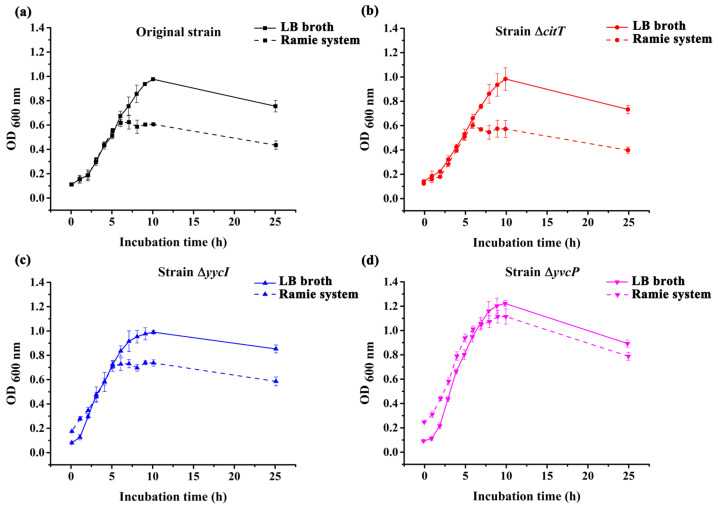
Growth curves of original and mutant strains in LB medium and ramie degumming system. Growth was monitored by measuring the OD_600 nm_ values. Curves represent the growth dynamics of the original strain and the Δ*citT*, Δ*yvcP*, and Δ*yycI* mutant strains in different culturing systems. (**a**) Growth curve of original strain; (**b**) Growth curve of strain Δ*citT*; (**c**) Growth curve of strain Δ*yycI*; (**d**) Growth curve of strain Δ*yvcP*.

Gene knockout is one of the effective methods for exploring the function of a specific protein. The successful knockout of the genes *citT*, *yvcP*, and *yycI* in the mutant strains Δ*citT*, Δ*yvcP*, and Δ*yycI* was confirmed by PCR analysis (Figure 2). Subsequently, the growth characteristics of the original strain and the mutant strains Δ*citT*, Δ*yvcP*, and Δ*yycI* were analyzed under LB culturing conditions and in the ramie degumming system by monitoring the absorbance at 600 nm (Figure 3). Interestingly, there was no significant difference in the duration of the lag phase, logarithmic growth phase, stationary phase, and decline phase among the four strains in both culturing systems. Moreover, in both the LB culturing system and the ramie degumming system, the strain with the *yvcP* gene knockout exhibited the highest biomass accumulation, while no significant differences in biomass accumulation were observed among the strains with *citT*, *yycI* gene knockouts and the original strain. However, whether the differences in cell concentration are with correlated to the degumming efficiency of ramie fibers remains to be further explored.

### 3.3. Identification of the Key TCS Response Regulator Proteins in CitT, YvcP, and YycI

**Figure 4:**
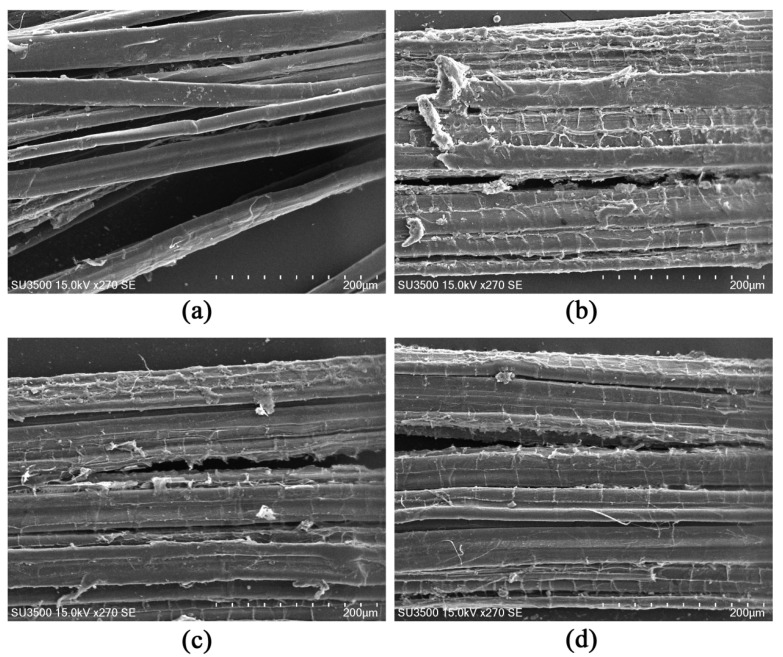
Scanning electron microscopy (SEM) images of ramie fibers after microbial degumming by original and mutant strains. (**a**) Degumming by original strain; (**b**) Degumming by Δ*citT* mutant strain; (**c**) Degumming by Δ*yvcP* mutant strain; (**d**) Degumming by Δ*yycI* mutant strain.

**Figure 5:**
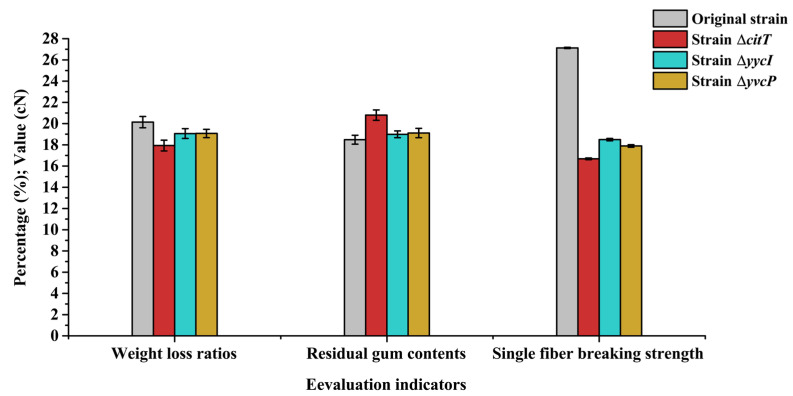
Evaluation of ramie fiber degumming efficiency by original and mutant strains. Weight loss ratios, residual gum contents, and single-fiber breaking strength of degummed ramie fibers were measured to assess the degumming performance of different strains.

**Figure 6:**
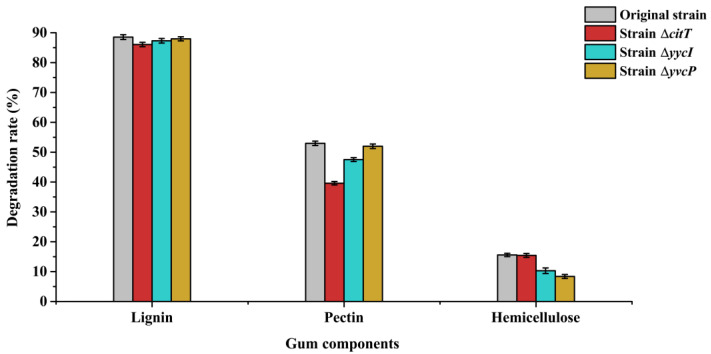
Analysis of polysaccharide degradation rates of gum components in ramie bast by original and mutant strains. Degradation rates of lignin, pectin, and hemicellulose were measured to evaluate the metabolic regulation of gum degradation by CitT, YvcP, and YycI in *B. subtilis*.

To investigate which of the three proteins, CitT, YvcP, and YycI, is the key TCS response regulator for the efficiency of ramie bast degumming, the surface morphology of degummed ramie fibers was examined using SEM (Figure 4). The results showed that the surface of ramie fibers degummed with the original strain was smooth, with minimal residual gum. In contrast, fibers degummed with strains lacking CitT, YvcP, or YycI proteins exhibited increased gum residues. Notably, SEM analysis revealed that the mutant strain lacking CitT exhibited significant accumulation of residual gum on the fiber surface during the degumming process (Figure 4). This morphological observation was consistent with the corresponding impairment in weight loss ratios, residual gum contents, and polysaccharide degradation rates (Figure 5 and Figure 6), suggesting that CitT plays a more critical role in regulating degumming efficiency compared to YvcP and YycI.

Furthermore, to provide additional evidence on the impact of CitT, YvcP, and YycI protein deficiencies on the degumming of ramie fibers, the performance of ramie fibers degummed by different strains was evaluated from three aspects: weight loss ratios, residual gum contents, and single fiber breaking strength (Figure 5). The results indicated that the absence of CitT, YvcP, and YycI proteins all reduced the degumming efficiency of *B. subtilis*, with the most significant impact observed for CitT protein, followed by YvcP protein, and the least impact for YycI protein.

### 3.4. Functional Analysis of Different Regulatory Proteins

As the results above show, the absence of proteins CitT, YycI, and YvcP all led to a decrease in the ramie degumming efficiency of *B. subtilis*. However, the specific roles these three proteins play in regulating the degradation of different gum components remain to be further elucidated. So, the degradation rates of lignin, pectin, and hemicellulose in ramie bast by degumming with the original strain and the three strains mutant Δ*citT*, Δ*yvcP*, and Δ*yycI* were measured in this study (Figure 6). The results showed that the absence of CitT, YycI, and YvcP proteins had no significant impact on lignin degradation rate, which may be attributed to the relatively low efficiency of bacterial lignin degradation as compared to fungi [41]. The absence of proteins CitT and YycI both reduced the degradation rate of pectin, with the absence of CitT protein having a more significant effect on the pectin degradation, the results was consistent with the previous studies [32,42]. The absence of proteins YycI and YvcP both decreased the degradation rate of hemicellulose, with the absence of YvcP protein having a greater impact on hemicellulose degradation. Two-component systems (TCS) are generally important for microbial regulation under conditions of osmotic, oxidative and nutritional stress [43]. The results of this study demonstrated that TCS plays a significant role in regulating the utilization of polysaccharides as carbon sources by *B. subtilis*. Moreover, the different TCS response regulators, CitT, YycI, and YvcP, exhibit distinct regulatory functions in the degradation of different types of polysaccharides.

**Figure 7:**
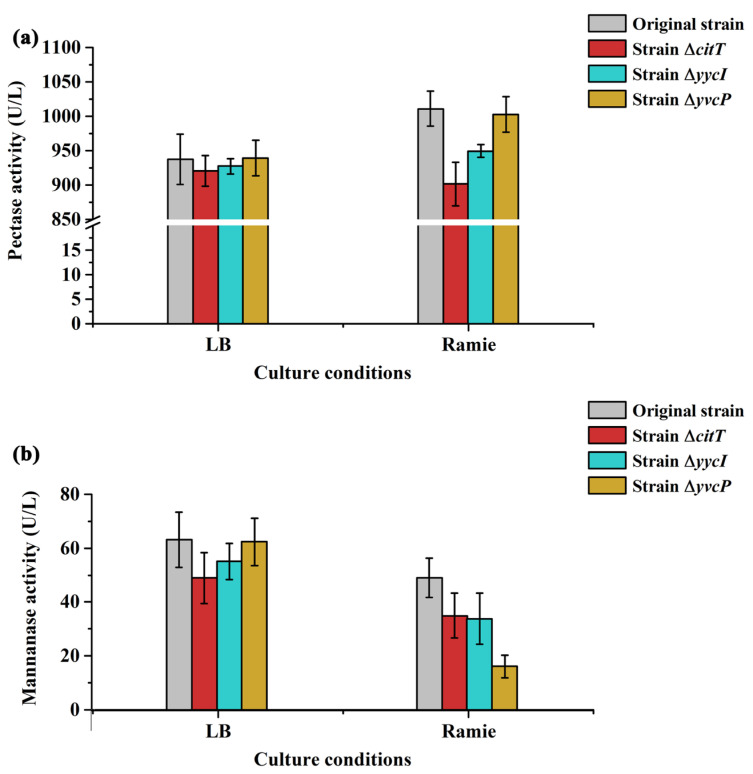
Pectinase and β-mannanase activities of the original and mutant strains under different culture conditions. (**a**) Pectinase activity; (**b**) β-mannanase activity.

**Figure 8:**
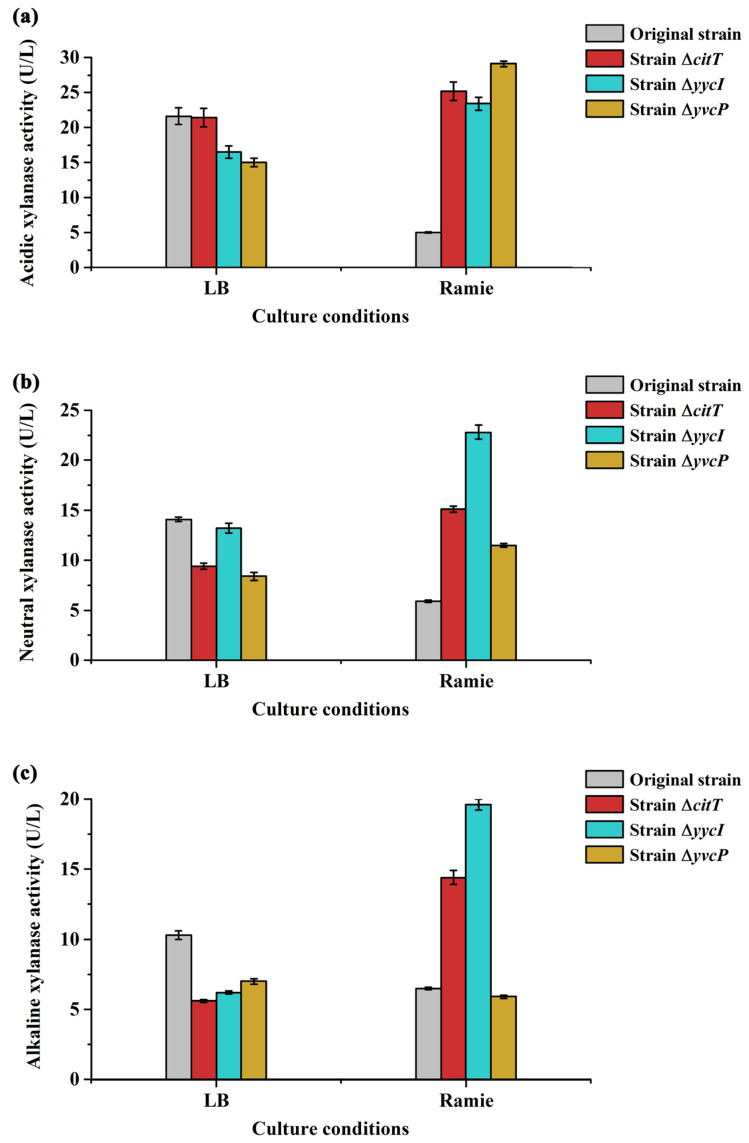
Xylanase activity of the original and mutant strains under different culture conditions. (**a**) Acidic xylanase activity; (**b**) Neutral xylanase activity; (**c**) Alkaline xylanase activity.

To further investigate the impact of knocking out *citT*, *yycI* and *yvcP* on enzymatic activity, the enzymatic profiles of both original and mutant strains cultivated in LB broth and the ramie bio-degumming system were quantitatively assessed and showed in Figure 7 and Figure 8. Although lignin is present in small amounts in ramie bast gum, pectin and hemicellulose constitute the major gum components [3]. Pectinase is widely recognized as the pivotal enzyme responsible for pectin degradation [13]. Similarly, xylanase and mannanase play crucial roles in the biodegradation of hemicellulose, with mannanase exhibiting a synergistic effect with xylanase during microbial utilization of hemicellulosic substrates [9]. Therefore, we focused on the activities of pectinase, β-mannanase, acidic xylanase, neutral xylanase, and alkaline xylanase—key extracellular enzymes involved in the bio-degumming of ramie fibers. The results revealed that within the ramie degumming system, the activities of pectinase and mannanase correlated well with the degradation rates of polysaccharides in the gum (Figure 7). Across both LB medium and the degumming system, the original strain exhibited the highest pectinase activity, while the CitT-deficient mutant showed the lowest. Notably, only the CitT-deficient mutant demonstrated higher pectinase activity in LB than in the ramie system, suggesting that the presence of ramie pectin—as a specific carbon source—failed to induce pectinase activity with the absence of CitT. This aligned with the observed degradation pattern of pectin (Figure 6), supporting the conclusion that CitT is a key transcriptional regulator controlling pectin degradation during ramie degumming. Interestingly, β-mannanase activity decreased across all strains when shifting from LB medium to the ramie degumming system, with the most pronounced reduction occurring in the YvcP-deficient mutant. This was consistent with the proposed role of YvcP in regulating hemicellulose degradation during the degumming process.

*B. subtilis* strain 168 produces four extracellular xylanases: XynA (encoded by *xynA*), β-xylosidase (XynB, encoded by *xynB*), glucuronoxylanase (XynC, encoded by *xynC*), and arabinoxylan arabinofuranohydrolase (XynD, encoded by *xynD*). According to previous reports, the optimal pH for XynA activity ranges from 6.0 to 6.5 [44], while that for XynC is between 5.0 and 6.5 [45]; the optimal pH for XynB and XynD remains unknown. Therefore, this study assessed the activities of acidic, neutral, and alkaline xylanases to comprehensively evaluate changes in xylanase activity within the ramie degumming system and its correlation with hemicellulose degradation. Notably, our analysis revealed that the original strain exhibited the lowest activities of acidic, neutral, and alkaline xylanases among all tested strains when cultured in the ramie degumming system. Moreover, all three types of xylanase activities decreased significantly when the original strain was switched from LB medium to the ramie system. We speculated that this discrepancy may be attributed to a substrate mismatch—specifically, the use of beechwood xylan as the substrate for enzymatic assays, which may not optimally reflect the activity of *B. subtilis* xylanases toward ramie-derived xylan [46]. It is plausible that using isolated ramie xylan could yield different trends, a hypothesis that warrants further investigation. Furthermore, previous studies on microbial degumming of kenaf suggested that xylanase activity does not exhibit a clear correlation with degumming efficiency [47]. This supports the possibility that xylanase activity may also play a limited role in the degumming efficiency and hemicellulose degradation during ramie processing.

## 4. Conclusions

This study systematically investigated the functions of three TCS response regulators—CitT, YvcP, and YycI—in the *B. subtilis*-mediated degumming of ramie fibers. The key findings are as follows: No protein–protein interactions were detected among CitT, YvcP, and YycI, suggesting that they regulate distinct metabolic pathways in *B. subtilis*. Gene knockout experiments revealed that the absence of these regulators differentially impaired degumming efficiency, with the CitT mutant exhibiting the most severe reduction in gum degradation, identifying CitT as the pivotal regulator in this process. Furthermore, functional analyses indicated that CitT primarily facilitates pectin degradation, YvcP is mainly involved in hemicellulose decomposition, and YycI participates in the degradation of both polysaccharides. These results provide novel insights into the polysaccharide-specific regulatory roles of TCS response regulators in *B. subtilis* and establish a genetic basis for constructing high-efficiency engineered strains for enhanced ramie degumming.

## Data Availability

The datasets generated during and/or analyzed during the current study are available from the corresponding author on reasonable request.
